# Decompressive craniectomy following traumatic brain injury: developing the evidence base

**DOI:** 10.3109/02688697.2016.1159655

**Published:** 2016-03-14

**Authors:** Angelos G. Kolias, Hadie Adams, Ivan Timofeev, Marek Czosnyka, Elizabeth A. Corteen, John D. Pickard, Carole Turner, Barbara A. Gregson, Peter J. Kirkpatrick, Gordon D. Murray, David K. Menon, Peter J. Hutchinson

**Affiliations:** ^a^ Division of Neurosurgery, Department of Clinical Neurosciences, Addenbrooke’s Hospital & University of Cambridge, Cambridge Biomedical Campus, Cambridge, UK; ^b^ Institute of Neuroscience, Neurosurgical Trials Group, Newcastle University, Newcastle, UK; ^c^ Centre for Population Health Sciences, University of Edinburgh, Edinburgh, UK; ^d^ Division of Anaesthesia, Addenbrooke’s Hospital & University of Cambridge, Cambridge Biomedical Campus, Cambridge, UK

**Keywords:** Acute subdural haematoma, brain oedema, clinical trial, intracranial pressure, traumatic brain injury

## Abstract

In the context of traumatic brain injury (TBI), decompressive craniectomy (DC) is used as part of tiered therapeutic protocols for patients with intracranial hypertension (secondary or protocol-driven DC). In addition, the bone flap can be left out when evacuating a mass lesion, usually an acute subdural haematoma (ASDH), in the acute phase (primary DC). Even though, the principle of “opening the skull” in order to control brain oedema and raised intracranial pressure has been practised since the beginning of the 20th century, the last 20 years have been marked by efforts to develop the evidence base with the conduct of randomised trials. This article discusses the merits and challenges of this approach and provides an overview of randomised trials of DC following TBI. An update on the RESCUEicp study, a randomised trial of DC versus advanced medical management (including barbiturates) for severe and refractory post-traumatic intracranial hypertension is provided. In addition, the rationale for the RESCUE-ASDH study, the first randomised trial of primary DC versus craniotomy for adult head-injured patients with an ASDH, is presented.

## Introduction

Traumatic brain injury (TBI) remains a major public health problem across the globe.^1^ Intracranial pressure (ICP) following TBI can be elevated due to increasing mass effect from haematomas and contusions, diffuse brain swelling or hydrocephalus. Intracranial hypertension can lead to ischemia due to reduction of the cerebral perfusion pressure (CPP). Substantial evidence from large cohort studies points to the fact that intracranial hypertension is associated with excess mortality following TBI.^2^ ^,^ ^3^ Decompressive craniectomy (DC) is a surgical procedure which involves removal of a large part of the skull and opening of the underlying dura mater. From a physiological viewpoint, DC helps to overcome the rigid and non-compliant nature of the skull and the dura mater, thereby leading to a reduction of the ICP.^4^ However, even though >100 years have passed since the first description of DC by Theodor Kocher in the 20th century, uncertainties still remain regarding the indications, optimal timing and effects of DC on the functional outcome of head-injured patients.^5^

## Definitions – primary and secondary DC

Primary DC refers to leaving a large part of the skull (bone flap) out after evacuating an intracranial haematoma (mass lesion) in the early phase after the head injury.^5^ ^,^ ^6^ Mass lesions can be extradural, subdural, intraparenchymal or a combination thereof.^7^ However, the most frequent indication for a primary DC is an acute subdural haematoma (ASDH).^8–10^ Typically, a large fronto–temporo-parietal bone flap (hemi-craniectomy) is left out after evacuating the haematoma either because the brain is bulging beyond the inner table of the skull or because there is a concern of increasing brain swelling (e.g. in a patient with contusions) in the post-operative period.^11^ ^,^ ^12^

A DC may also be undertaken in head-injured patients who are managed in an intensive care unit (ICU) with ICP monitoring. This is usually referred to as a secondary DC.^5^ ^,^ ^6^ In this context, a DC is performed as part of tiered therapeutic protocols which aim to control raised ICP and ensure adequate CPP.^13^ The operation can be undertaken as last-tier (life-saving) therapy when all other measures have failed to reduce ICP at levels <25–35 mmHg. Alternatively, it can be performed as a second-tier therapy in patients with less pronounced elevation of ICP (e.g. 20 mmHg); this can be viewed as a neuro-protective measure.^5^ Three main options exist in terms of the site of a secondary DC: bifrontal (bone flap extends from the floor of the anterior cranial fossa anteriorly to the coronal suture posteriorly and to the middle cranial fossa floor bilaterally), hemi-craniectomy (same as described earlier) and bilateral hemi-craniectomy.

## Decompressive craniectomy in the 20th century

The first modern report of the use of DC following TBI was published by Harvey Cushing in 1908.^14^ Cushing treated head-injured patients with a subtemporal DC and he reported a substantial reduction in mortality. The period from the 1950s to 1970s was marked by divided opinions regarding the utility of DC.^5^ Proponents of DC were calling for controlled studies but the opponents focused their criticism on the complications of DC and the fact that patients surviving after a DC may be left in a severely disabled or vegetative state. The management of TBI progressed significantly in the 1980s and 1990s due to advances in neuroimaging (widespread introduction of CT scanning), prehospital management, neurointensive care (widespread adoption of ICP monitoring and tiered therapeutic protocols) and rehabilitation. This led to a renaissance of interest in DC with many uncontrolled studies reporting a survival benefit with DC.^15^ ^,^ ^16^

## Decompressive craniectomy in the 21st century – developing the evidence base

**Figure 1.**
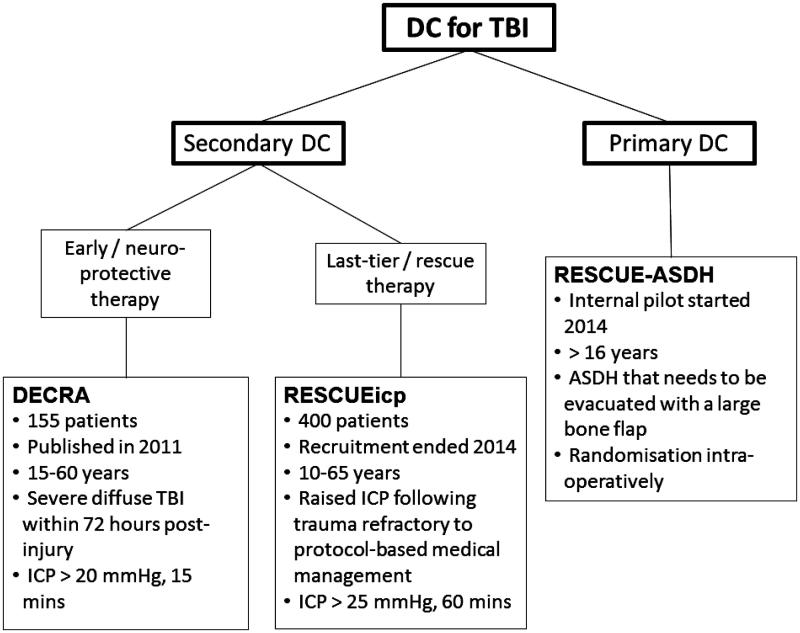
Randomised trials of decompressive craniectomy for TBI.

The 21st century, so far, has seen consistent efforts to improve the evidence base for DC following TBI with the conduct of randomised trials (Figure 1). At this point, we need to consider what evidence based medicine is and why it is necessary. Evidence based medicine has been defined as “the conscientious, explicit and judicious use of current best evidence in making decisions about the care of individual patients”.^17^ Sackett et al. have described best available external clinical evidence as “clinically relevant research, often from the basic sciences of medicine, but especially from patient centred clinical research into the accuracy and precision of diagnostic tests (including the clinical examination), the power of prognostic markers, and the efficacy and safety of therapeutic, rehabilitative and preventive regimens”.^17^ It is desirable, but not always possible, for such evidence to be of high quality, with good internal and external validity, low risk of bias and reproducibility. The same authors have also suggested that “external clinical evidence can inform, but can never replace, individual clinical expertise, and it is this expertise that decides whether the external evidence applies to the individual patient at all and, if so, how it should be integrated into a clinical decision”.^17^

Experimental study designs can provide the evidence needed to answer pertinent clinical questions. To study the efficacy of a treatment, there needs to be a control group, ideally in the context of a randomised controlled trial (RCT). In this setting the allocation of individuals to the treatment or control group occurs randomly. Because of the random assignment the treatment and control group are balanced, both in terms of observable and non-observable characteristics. Therefore differences in the outcomes between the two groups are attributed to the experimental effect of exposure to the treatment. However, RCTs are sometimes not feasible due to practical or ethical reasons. Moreover, RCTs are not necessary for the evaluation of treatments with dramatic effects that are unlikely to have resulted from inadequately controlled biases. In addition, a number of factors, such as lack of clinical equipoise, strong patient preferences, imbalance in surgical expertise, poor compliance with allocated treatment (cross-over), difficulty with blinding render trials of surgical interventions particularly challenging.^18^ Trials in severe TBI patients face a number of additional complexities; clinical research in the emergency/ICU setting is difficult, patients often lack the capacity to consent, care of severe TBI is multifaceted and clinicians often have strong views especially when comparing surgical with medical therapies. In addition, new approaches, such as comparative effectiveness research (CER) have gained traction in recent years. Non-experimental CER studies – such as the CENTER-TBI project (https://www.center-tbi.eu/) – aim to utilise the heterogeneity in systems, practices and outcomes in order to compare the effectiveness of interventions that are standard practice in some centres but not in others.^19^ These efforts are extremely important and promising and should be seen as being complementary to randomised trials. Pragmatic RCTs – that aim to compare two or more treatments in the “real-world” – are a form of CER, the so-called experimental CER. We regard RCTs and non-experimental CER, as two equally important facets of TBI research aiming to answer questions regarding the clinical effectiveness of interventions. At the same time, RCTs are widely accepted as the gold-standard method for assessing the efficacy and effectiveness of therapeutic interventions and, although difficult, surgical evaluation is achievable and necessary. Hence, we believe that when a research question is sufficiently refined to allow the design of an RCT, one should be undertaken if feasible.

### BEST-TRIP trial

This study was a multi-centre randomised trial that included a total of 324 patients with severe TBI treated in ICUs in Bolivia and Ecuador.^20^ The study compared management guided by ICP monitoring aiming to maintain ICP at or below 20 mmHg with management guided by clinical examination and serial computed tomography (CT) imaging. Hence, it should be emphasised that BEST-TRIP was not a trial of ICP monitoring and did not seek to determine the efficacy of ICP monitoring. Even though a difference in the outcomes between the two arms was not found, since the publication of the study, several editorials and study correspondence have pointed out limitations in the trial design that limit the external validity and generalisability of its findings.^21^ ^,^ ^22^ Importantly, patients in both arms received tiered ICP-lowering therapies, and 30% of patients in each arm received a DC. The BEST-TRIP trial, therefore, does not challenge the fundamental concept that brain oedema and raised ICP should be actively managed in patients with TBI.^5^

### DECRA trial

The DECRA study was conducted between 2002 and 2010 in Australia, New Zealand and Saudi Arabia.^23^ The study recruited 155 patients with severe diffuse TBI and moderate intracranial hypertension. Patients were randomised within the first 72 h following TBI if their ICP exceeded 20 mmHg for >15 min – continuously or intermittently) within a 1-h period, and if they did not respond to optimised first-tier ICP-lowering interventions. The two arms of the trial were bifrontal DC and standard medical management or standard medical management alone. Patients in the surgical arm were found to have better ICP control, received fewer interventions for raised ICP, and had a reduced length of stay at the ICU. However, the investigators observed a higher rate of unfavourable outcomes in surgical patients (70% versus 51%; OR 2.21; 95% CI 1.14–4.26; *p* = 0.02). Nevertheless, 27% of patients in the surgical arm had bilaterally unreactive pupils compared with only 12% in the medical arm. A *post hoc* adjustment for pupil reactivity at baseline, which was necessary as pupil reactivity is known to be a major prognostic indicator of outcome following TBI, revealed that the rate of unfavourable outcome was not significantly different between the two arms (adjusted OR 1.90; 95% CI 0.95–3.79). Overall, the DECRA study provided convincing evidence that early neuro-protective bifrontal DC is not superior to medical management for patients with diffuse TBI.

### RESCUEicp trial

In contrast to the DECRA study, the RESCUEicp trial is examining the effectiveness of DC (bifrontal or unilateral) as a last-tier therapy for patients with severe, sustained and refractory post-traumatic intracranial hypertension.^24^ Patients were randomised to DC or continuing medical therapy if their ICP exceeds 25 mmHg for at least 1 h and is not responding to tiered ICP-lowering therapies. A barbiturate infusion is not allowed pre-randomisation; it only becomes an option for patients randomised in the medical arm following randomisation. RESCUEicp is also a multi-centre study but has a number of important differences from DECRA: sample size (400 patients in RESCUEicp versus 155 patients in DECRA); surgical technique (bifrontal or unilateral DC versus bifrontal DC only); ICP threshold (25 mmHg versus 20 mmHg); duration of refractory intracranial hypertension (at least 1 h versus 15 min) and timing of randomisation (any time when inclusion criteria are met versus within 72 h after TBI only).^25^ Enrolment of new patients to the RESCUEicp trial ended on 31 March 2014 and the study is currently in follow-up.

The Statistical Analysis Plan (SAP) was agreed without reference to the unblinded data, approved by the independent chair of the Trial Steering Committee and released in the public domain in October 2015. The SAP can be found in the Supplementary material. The results (primary endpoint) are expected in 2016.

## RESCUE-ASDH trial

Two-thirds of TBI patients undergoing emergency neurosurgery have an ASDH evacuated. These hematomas have been associated with a high mortality rate and low rates of functional recovery.^11^ In addition, parenchymal injuries – such as contusions – and brain swelling are often found in patients with ASDH.^8^ Miller et al. reported that two-thirds of the 48 patients with an evacuated ASDH had raised ICP in the post-operative period; increased ICP was defined as persistent elevation of mean ICP >20 mmHg during the period of continuous monitoring of ICP in the ICU.^26^ Importantly, half of the patients with raised ICP developed uncontrollable intracranial hypertension leading to herniation and death. Wilberger et al. observed that 40% of their cohort of 101 comatose patients who underwent a craniotomy for an ASDH had an ICP which remained <20 mmHg in the post-operative period, while 43% had a sustained period of uncontrollable intracranial hypertension with ICP peaking >45 mmHg.^27^ Importantly, the authors observed that the mortality rate was ∼40% in the former but close to 95% in the latter subgroup. These studies provide convincing evidence that, firstly, ICP can be elevated after ASDH evacuation and, secondly, elevated ICP leads to higher mortality.

In a retrospective cohort comparison study of 91 patients who had an operation for an ASDH, 56% received a primary DC, while the rest a craniotomy.^10^ The standardised morbidity ratio was lower in patients who had a DC (0.75; 95% CI 0.51–1.07) compared to those who had a craniotomy (0.90; 95% CI 0.57–1.35). Although the 95% confidence intervals overlap, this study supports the hypothesis that a primary DC (i.e. bone flap left out after ASDH evacuation) may lead to better outcomes compared to a craniotomy (i.e. bone flap is replaced) due to better control of brain swelling and intracranial hypertension in the post-operative period. This hypothesis is also supported by a recently published two-centre non-experimental CER study, which found that post-operative ICP was better controlled and patient outcomes were better in the centre with greater utilisation of primary DC.^9^

However, there is a paucity of high-quality evidence in the literature regarding the best surgical strategy (primary DC or craniotomy) for this group of patients and surgical decision making is often haphazard.^28^ In a survey of UK surgeons, a significant variation in the surgical management of ASDH was observed with 41% of the respondents using primary DC <25% of the time but approximately one-third using DC in >50% of such cases.^29^

On this background, the RESCUE-ASDH study was funded by the UK National Institute for Health Research (NIHR) as a multi-centre, pragmatic, parallel group randomised trial that aims to compare the clinical and cost-effectiveness of primary DC versus craniotomy for the management of adult head-injured patients undergoing evacuation of an ASDH. The trial was designed as a collaborative effort involving members of the British Neurosurgical Trainee Research Collaborative (BNTRC; www.bntrc.org.uk) and British Neurotrauma Group (BNTG; www.ukneurotrauma.org.uk), clinicians and academics with an interest in TBI, members of the Cambridge Clinical Trials Unit, health economists and service user representatives.

The criteria which are being used to determine eligibility of individual patients are:

- Inclusion criteria:
  - Adult head-injured patients (>16 years)
  - ASDH on CT*
  - The admitting neurosurgeon feels that the haematoma needs to be evacuated either by a craniotomy or DC (bone flap at least 11 cm in both instances)*

* Patients with additional lesions (e.g. intracerebral haemorrhage, contusions) can be included

- Exclusion criteria:
- Bilateral ASDHs both requiring evacuation
- Previous enrolment in RESCUE-ASDH study
- Severe pre-existing physical or mental disability or severe co-morbidity which would lead to a poor outcome even if the patient made a full recovery from the head injury.

Eligible patients are randomised to craniotomy or DC intra-operatively after evacuating the ASDH. Patients with significant brain swelling preventing safe replacement of the bone flap are not suitable for randomisation and are being followed-up in the context of an observational cohort.

The primary outcome measure is the extended Glasgow Outcome Scale (GOSE) at 12-month post-injury (Table 1). The secondary outcome measures are:

**Table 1.** The eight categories of the extended Glasgow Outcome Scale (GOSE).

Score	Category
1	Death
2	Vegetative state
3	Lower severe disability
4	Upper severe disability
5	Lower moderate disability
6	Upper moderate disability
7	Lower good recovery
8	Upper good recovery

- GOSE at 6 months
- quality of life (EQ-5D) at discharge from neurosurgical ward, 6 and 12 months
- Glasgow Coma Scale (GCS) on discharge from the intensive care unit (ICU) and from neurosurgical ward
- length of stay in ICU, neurosurgical and rehabilitation unit
- discharge destination from acute neurosurgical ward
- serious adverse events and surgical complications
- subsequent complications/re-admissions within the 1-year follow-up period
- return to operating theatre for cranial surgery within 2 weeks after randomisation
- incidence of hydrocephalus
- therapy intensity level in the post-intervention period
- economic evaluation

Analysis will be performed on an “intention-to-treat” basis with a proportional odds model adjusted for covariates. Retrospective studies suggest a favourable outcome (moderate disability or good recovery) in ∼35% of patients undergoing evacuation of ASDH.^11^ The sample size of 990 patients (495 in each arm; 10% drop out rate) will allow us to detect the equivalent of an 8% absolute difference in favourable outcome [90% power and two-sided significance 0.05 (35% versus 43%)]. This corresponds to the equivalent of an 8% treatment effect.

The internal pilot phase of the study started in UK in autumn 2014. We are now just over 1 year since recruitment started and the study has achieved the following milestones (as of 29th November 2015):

- 64 patients have been randomised from 15 UK sites.
- 57 patients have been enrolled in the observational study cohort.
- 21 centres are now open to recruitment in UK and 2 more in set up.

Clinicians interested to collaborate are encouraged to visit http://www.rescueasdh.org/contact-us for further information.

## Conclusions

The 21st century has been marked by efforts to develop the evidence base for DC following TBI. Current evidence suggests that early (neuroprotective) bifrontal DC is not superior to medical management for patients with diffuse TBI. Secondary DC as a last-tier therapy for severe and refractory post-traumatic intracranial hypertension is the subject of the RESCUEicp study, while primary DC for patients with ASDH is being systematically evaluated in the context of the RESCUE-ASDH trial. In view of the currently available evidence, and owing to the number of potential complications with DC, indiscriminate use of DC for patients with TBI is not appropriate.

## Supplementary Material

Supplementary_material_BJN_1159655.pdf
