# Supplementary material for: Decompressive craniectomy following traumatic brain injury: developing the evidence base
Source: Br J Neurosurg. 2016 Mar 14;30(2):246–50. doi: 10.3109/02688697.2016.1159655 (PMC4841020; doi:10.3109/02688697.2016.1159655)
Supplement: Supplementary_material_BJN_1159655.pdf [file ibjn_a_1159655_sm7220.pdf]

Statistical Analysis Plan for the RESCUEicp Trial

Randomised Evaluation of Surgery with Craniectomy for Uncontrollable Elevation of Intra-Cranial Pressure (RESCUEicp)

ISRCTN 66202560

Statistical Analysis Plan Version 1.3

16th September 2015

Author: Gordon Murray, Trial Statistician

1.0 INTRODUCTION

The RESCUEicp trial protocol (Version 6, dated August 2015) describes the study design. The purpose of this Statistical Analysis Plan is to give a more detailed and comprehensive description of the methods for the analysis of the trial data, to avoid post hoc decisions that may affect the interpretation of the results of the statistical analysis.

2.0 RESCUEicp PROTOCOL

RESCUEicp is a randomised trial comparing optimal medical management with surgery (decompressive craniectomy) for the management of intra-cranial hypertension following head injury, refractory to first-line and second-line treatment measures (as per flowchart in page 15 of the protocol).

The study design is a two arm parallel group open randomised trial with a target total sample size of 400 patients (i.e. two groups of 200 patients) randomised to receive either optimal medical management or decompressive craniectomy.

2.1 Clinical Outcome Measures as Specified in the Trial Protocol (Version 6, dated August 2015)

1. The primary endpoint is the Extended Glasgow Outcome Scale (GOSE) at 6 months after randomisation.

2. The secondary endpoints are:
    a. GOSE at 12 and 24 months after randomisation
    b. Mortality at 6, 12 and 24 months after randomisation
    c. SF-36 and SF-10 (below 16 years) questionnaires at 6, 12 and 24 months after randomisation
    d. Glasgow Coma Scale (GCS) at discharge from neurosciences hospital
    e. Assessment of ICP control
    f. Time in intensive care
    g. Time to discharge from the neurosciences hospital
    h. Detailed health-economic analysis

Adverse events will also be reported.

2.2 Statistical Considerations

Sample size determination: The total number of patients will be 400 (200 in each arm of the study) for a 15% difference in outcome (increase in favourable outcome from 45% to 60%) (power 80%, 2-sided significance 0.05, loss to follow-up up to 15%).

Randomisation: Participants were randomly assigned to either surgical or medical therapy with a 1:1 allocation as per a computer generated randomisation schedule using permuted blocks of random sizes and stratified

by site. The block sizes were not disclosed in order to ensure concealment. Participants were randomised using a central telephone randomisation service. Allocation concealment was ensured, as the service did not release the randomisation code until the patient had reached stage 3 of the protocol.

3.0 SCOPE OF THIS STATISTICAL ANALYSIS PLAN (SAP)

This SAP covers the analysis of the clinical endpoints of the trial up until the 12 month assessment. The trial will be analysed and reported when the data to 12 months are available for analysis. Subsequent analysis of data from the 24 month assessment will be performed using the same statistical approach. A separate health-economic analysis plan will cover the analysis of the SF-36/SF-10 data and other health-economic endpoints.

4.0 DETAILED ANALYSIS PLAN

4.1 Analysis populations

The analysis of all outcome (efficacy/effectiveness) measures and adverse events will be performed on the intention to treat (ITT) population, which will be defined as all randomised patients. The only exclusions will be those patients where the outcome data are not available and/or where consent was withdrawn.

A sensitivity analysis will be performed for the primary outcome measure using the per protocol (PP) population. The PP population will be defined as those patients included in the ITT population but excluding any patients where there had been a severe breach of protocol. Such exclusions will be agreed by the Trial Steering Committee ahead of the database being locked and unblinded, and will include, for example, patients where ICP was not monitored.

4.2 Primary Outcome Measure

The primary outcome measure will be the extended Glasgow Outcome Scale (GOSE) evaluated at six months. The main analysis will be an ordinal analysis based on the proportional odds model, with the result presented as the estimated common odds ratio with its corresponding 95% confidence interval and p-value. For this primary analysis the GOSE categories of upper good recovery and lower good recovery will be pooled since a blinded review of the distribution of GOSE revealed that there are too few patients in these categories to allow them to be analysed as separate categories.

The goodness-of-fit of this unadjusted proportional odds model will be tested. If the proportional odds model is rejected at the 5% significance level then that in itself will mean that there is evidence of a difference in the distribution of the GOSE scores between the two randomised groups, and the interpretation of the results will focus on describing the difference.

As a sensitivity analysis the proportion of patients achieving a ‘favourable’ outcome (defined as upper severe disability or better on the GOSE scale) will be compared between the randomised groups using a chi-squared test. The result will be presented as the estimated odds ratio with its corresponding 95% confidence interval and p-value, for comparison with the estimated common odds ratio, and also as the estimated absolute difference in the proportions with the corresponding 95% confidence interval.

Further exploratory analyses will examine the impact of covariate adjustment on the above analyses using ordinal and binary logistic regression respectively. Age, GCS motor score, pupils, and the last available pre-randomisation Marshall CT class will be taken as covariates. Three separate analyses will be undertaken as the GCS motor score and pupils have been recorded at three time points (initial, on arrival at the first hospital, on arrival at the neurosciences hospital). Mean/median value imputation will be used to allow the small proportion of patients with missing values for their baseline covariates to be included in these analyses.

The Glasgow Coma Scale sum at discharge will be analysed using the same ordinal methodology described above for the GOSE. If necessary, adjacent categories will be pooled if there are too few patients with any particular GCS.

4.3 Secondary Outcome Measures

ICP control
The following will be presented:
a. Mean ICP after randomisation (in mmHg)
b. Number of hours with ICP > 25 mmHg after randomisation
c. Intracranial hypertension index. This is the number of end-hourly measures of ICP > 20 mmHg divided by the total number of measurements, multiplied by 100. The number of end-hourly measures of ICP > 25 mmHg divided by the total number of measurements, multiplied by 100 will also be presented.
d. Cerebral hypoperfusion index. This is the number of observations of cerebral perfusion pressure of less than 60 mm Hg divided by the total number of measurements, multiplied by 100. Cerebral perfusion pressure is the mean arterial pressure minus the intracranial pressure.

Duration of intensive care and duration of neurosurgical unit stay will be analysed using survival techniques, with Kaplan-Meier survival estimates and logrank tests to compare optimal medical management with surgery (decompressive craniectomy). Results will be presented as unadjusted hazard ratios with corresponding 95% confidence intervals and p-values.

GOSE at 12 months will be analysed using the same statistical methods set out above for the GOSE at 6 months.

4.4 Subgroup Analyses

There are six *a priori* proposals to perform subgroup analysis, namely:

- Diffuse injury (Marshall 1-4 on pre-randomisation CT) versus non-diffuse injury (Marshall 5-6 on pre-randomisation CT)
- Initial GCS 3-8 vs 9-15
- Age ≤ 40 versus age >40 years
- Craniotomy for evacuation of mass lesions before randomisation; yes versus no
- Time from injury to randomisation ≤ 72 versus > 72 hours
- Patients randomised in the UK versus rest of the world

These analyses will be performed by including a treatment by subgroup interaction term in the regression model relating 6 month GOSE to randomised treatment allocation and subgroup type.

4.5 Adjustment for multiplicity

Adjustment for multiplicity is considered unnecessary since the trial has a single pre-specified primary outcome measure. The secondary outcomes are exploratory and the results will only be interpreted as supportive evidence related to the primary outcome.

4.6 Adverse Events

The number and type of adverse events that were observed during the trial will be summarised separately for each treatment group, based on all randomised patients.
